# Preoperative anxiety state is an independent risk factor for delayed gastric emptying after pylorus-preserving pancreaticoduodenectomy: a single-center retrospective case-control study

**DOI:** 10.1080/07853890.2025.2564278

**Published:** 2025-09-23

**Authors:** Zhengwang Wang, Yuan Chen, Xinyu Ge, Ke Zhang, Jie Zhu, Peng Wang, Peng Xu, Jie Yao

**Affiliations:** ^a^ Department of Hepatobiliary and Pancreatic Surgery, Northern Jiangsu People’s Hospital Affiliated to Yangzhou University, Jiangsu Province, China; ^b^ Department of Hepatobiliary and Pancreatic Surgery, The Yangzhou School of Clinical Medicine of Dalian Medical University, Jiangsu Province, China; ^c^ Department of Hepatobiliary and Pancreatic Surgery, Northern Jiangsu People’s Hospital, Jiangsu Province, China

**Keywords:** Pylorus-preserving pancreaticoduodenectomy, delayed gastric emptying, anxiety state, risk factor analysis

## Abstract

**Background:**

Delayed gastric emptying (DGE) is one of the prevalent complications following pylorus-preserving pancreaticoduodenectomy (PPPD). Anxiety is one of the common psychological comorbidities among tumor patients. However, its influence on the development of DGE post-PPPD remains elusive. Thus, this study aimed to explore the relationship between anxiety states and the incidence of DGE after PPPD.

**Methods:**

This single-center, retrospective case-control study enrolled 150 patients who underwent PPPD between January 2020 and December 2022. Preoperative anxiety state was assessed using the Hamilton Anxiety Scale (HAMA), and patients were stratified into two groups based on the presence or absence of DGE. Lastly, risk factors for DGE were analyzed based on clinical data, laboratory indicators, postoperative complications, and other indicators.

**Results:**

The incidence of postoperative DGE was 28.67%. Patients who developed DGE had significantly higher body mass index (BMI) (23.50 ± 3.85 vs 22.20 ± 2.39, *p* = 0.013), a higher prevalence of hypertension (46.51% vs 28.97%, *p* = 0.040) and anxiety state (72.09% vs 48.60%, *p* = 0.009), as well as high preoperative blood lipid levels [1.87 (1.30, 2.36) vs 1.39 (0.96, 2.02), *p* = 0.005] may increase the occurrence of DGE. In contrast, high albumin levels on the third day postoperatively (33.18 ± 4.31 vs 35.23 ± 3.47, *p* = 0.003) conferred protective effects against DGE. Anxiety state (OR: 2.605, 95%CI: 1.170–6.138, *p* = 0.019) and high BMI (OR: 1.157, 95%CI: 1.022–1.332, *p* = 0.021) were identified as independent risk factors for DGE. After propensity score matching, anxiety state (*p* = 0.042) remained an independent risk factor for DGE.

**Conclusion:**

Anxiety state, BMI, hypertension, preoperative blood lipid levels, and albumin level on the third day after surgery were related to the occurrence of DGE after PPPD. Among them, anxiety state was identified as an independent risk factor for DGE. These findings highlight the need for implementing timely and appropriate psychological interventions in patients undergoing PPPD to minimize the risk of postoperative DGE.

**Trial Registration:**

ClinicalTrials.gov Identifier: ChiCTR2400090370.

## 1. Introduction

As is well documented, pancreatic cancer (PC) is one of the most prevalent malignancies worldwide and is characterized by aggressive behavior, a low surgical resection rate, and a poor prognosis. According to epidemiological studies, PC is the fourth leading cause of cancer death in the United States, with its mortality rate steadily rising [1]. It is projected to become the second leading cause of cancer death in the United States by 2030 [2]. Pancreatic head cancer is the most common type of PC, accounting for about 80% of all cases. Ampullary tumors arise from the pancreatic duct, distal bile duct, ampulla, or duodenum. The clinical symptoms, diagnosis and treatment of ampullary tumors are identical to those of pancreatic head cancer. Nevertheless, the prognosis is marginally more favorable than that of pancreatic head cancer, which may be ascribed to differences in pathogenesis or earlier clinical presentation [3]. In addition, with advances in diagnostic modalities and imaging techniques, the incidence of ampullary tumors is increasing annually [4].

In recent years, despite rapid advancements in chemotherapy, targeted therapy, and immunotherapy, pylorus-preserving pancreaticoduodenectomy (PPPD) remains the gold-standard treatment for pancreatic head cancer and ampullary tumors, and remains also the only potential curative treatment approach. Moreover, PPPD is also applied for the management of some benign diseases and borderline tumors in the proximal pancreas [5–7]. However, PPPD is a challenging surgical procedure with high postoperative risks and complication rates ranging between 30% and 50% [8].

Common complications after PPPD include bleeding, abdominal infection, diarrhea, anastomotic leakage, and gastrointestinal dysfunction. Besides, delayed gastric emptying (DGE) is the most common gastrointestinal dysfunction. Notably, DGE is a functional gastric emptying disorder that is not attributable to pharmacological agents or organic lesions in the stomach. Its incidence ranges between 20% and 40% [9]. PPPD preserves the pylorus compared to traditional pancreaticoduodenectomy (PD), thereby limiting the incidence of postoperative dumping syndrome and concurrently improving the quality of life and nutritional status of patients. Early studies have reported that PPPD increases the occurrence of DGE, whereas others have suggested a decreased risk [10–12]. It is worthwhile emphasizing that the pathogenesis of DGE is multifactorial. With the development of the biopsychosocial medical model, the impact of patients’ psychological state on disease treatment and prognosis has garnered widespread attention. Anxiety has been reported to be a common psychological comorbidity among cancer patients [13]. Previous studies have described that cancer patients with psychological disorders are associated with diminished quality of life and excessive psychological burden, which affects clinical outcomes [14,15]. However, studies investigating the relationship between anxiety and PC are scarce.

Consequently, this study retrospectively analyzed the incidence of DGE in 150 patients who underwent PPPD treatment at our center from January 2020 to December 2022. Patient history, past medical and surgical history, laboratory indicators, and postoperative complications were assessed, while the Hamilton Anxiety Scale (HAMA) was employed to evaluate preoperative anxiety status, to identify risk factors for DGE and to explore the influence of anxiety on the occurrence of DGE after PPPD.

## 2. Patients and methods

This study was conducted in accordance with the STROBE guidelines [16] and the Code of Ethics of the World Medical Association (Declaration of Helsinki). Ethical approval for this study was obtained from the Medical Ethics Committee of Northern Jiangsu People’s Hospital (approval number: 2024ky262). Written informed consent was obtained from all patients included in the study. This study was registered in the Chinese Clinical Trial Registry (registration number: ChiCTR2400090370).

### 2.1. Patients

This study retrospectively collected the data of patients with pancreatic head cancer, duodenal adenocarcinoma, bile duct adenocarcinoma, and other conditions (including tubulovillous adenoma of the duodenum, duodenal gastrointestinal stromal tumor, duodenal neuroendocrine tumor, mucoepidermoid carcinoma of the pancreas, pancreatic neuroendocrine tumor, and solid pseudopapillary tumor of the pancreatic head, hereafter referred to as ‘other diseases’) who underwent PPPD at our institution between January 2020 and December 2022 from the electronic medical record system. Preoperative indicators collected included age, gender, body mass index (BMI), history of smoking and alcohol consumption, hypertension, diabetes, prior surgical history, preoperative hemoglobin level, albumin level, triglyceride level, cholesterol level, bilirubin level, receipt of preoperative biliary drainage surgery, and presence of anxiety. Intraoperative indicators comprised operative time, intraoperative blood loss volume, and intraoperative blood transfusion. Postoperative indicators included tumor histopathological types, hemoglobin and albumin levels on postoperative days 1, 3, and 5, and the occurrence of DGE. All patients had a confirmed pathological diagnosis.

### 2.2. Inclusion and exclusion criteria

#### 2.2.1. Inclusion criteria

Patients with pancreatic head cancer, ampullary tumor and other diseases who were assessed and required PPPD following admission were enrolled.

#### 2.2.2. Exclusion criteria

1) Patients with pre-existing medical conditions that may cause gastroparesis, such as hypothyroidism; 2) Presence of mechanical gastrointestinal obstruction and gastroparesis prior to surgical intervention; 3) Patients with incomplete data.

### 2.3. Preoperative anxiety symptom score

Two trained assessors prospectively evaluated patients undergoing PPPD during the medical management process, and HAMA [17] was used to evaluate the presence of anxiety symptoms. According to the criteria established by the China Scale Collaboration Group, a total score <7 was considered as the absence of anxiety symptoms, whereas a total score ≥7 was indicative of the presence of anxiety symptoms.

### 2.4. Definitions of complications

The diagnostic criteria for postoperative complications following PPPD were based on the Guideline for the Prevention and Treatment of Common Complications after Pancreatic Surgery (2022) and the Clavien-Dindo classification system [18–20]. DGE was defined as the need for nasogastric intubation for more than 3 days, reinsertion of a nasogastric tube due to vomiting after extubation, or the inability to tolerate solid food by postoperative day 7. According to the International Study Group of Pancreatic Surgery (ISGPS) [21], DGE is classified into three grades: Grade A: Nasogastric intubation required for 4–7 days, or reinsertion of a nasogastric tube after removal on or after postoperative day 3, or inability to tolerate solid food by postoperative day 7, which may be accompanied by vomiting and/or the use of prokinetic drugs. Grade B: Nasogastric intubation required for 8–14 days, or reinsertion of a nasogastric tube after removal on or after postoperative day 7, or inability to tolerate solid food by postoperative day 14, accompanied by vomiting and/or the use of prokinetic drugs. Grade C: Nasogastric intubation required for more than 14 days, or reinsertion of a nasogastric tube after removal on or after postoperative day 14, or inability to tolerate solid food by postoperative day 21, accompanied by vomiting and/or the use of prokinetic drugs. Postoperative pancreatic fistula (POPF) was defined as drain fluid amylase concentrations exceeding three times the upper limit of normal serum amylase levels on or after postoperative day 3, accompanied by clinical symptoms. Bile leakage was defined as drain fluid bilirubin concentrations exceeding three times the upper limit of normal serum bilirubin levels on or after postoperative day 3, or the presence of biliary peritonitis requiring interventional or surgical management. Postoperative bleeding was defined as bloody fluid discharge *via* abdominal drainage tubes or nasogastric tubes, hematochezia, a significant drop in postoperative hemoglobin levels, or the need for further interventions such as blood transfusion, fluid resuscitation, interventional procedures, or surgical reoperation. Abdominal infection was diagnosed when fever and chills persisted for ≥24 h on or after 3 days postoperatively, accompanied by markedly elevated white blood cell count, procalcitonin, and high-sensitivity C-reactive protein levels, with imaging-confirmed intra-abdominal fluid accumulation. Definitive diagnosis was confirmed by positive bacterial cultures from abdominal drainage fluid.

### 2.5. Statistical analysis

Statistical analyses were performed using IBM SPSS Statistics (Version 26). One-to-one Propensity score matching (PSM) was conducted using Zstats 1.0 (www.zstats.net). Patients within the DGE group were matched to patients from the non-DGE group *via* nearest neighbor matching with a caliper width of 0.2 times the pooled standard deviation of the logit of the propensity score. Continuous variables following a normal distribution were expressed as mean ± standard deviation ($\bar{x}$ ±* s*) and compared using the Student’s *t*-test, whereas non-normally distributed variables were presented as median and interquartile range and compared using the Mann–Whitney *U* test. Categorical data were expressed as percentages (%) and compared using the Chi-square test or Fisher’s exact test. Multivariate analysis was carried out using Firth’s penalized likelihood logistic regression method *via* RStudio software (version R-4.5.1) with the following packages: logistf, readxl, car, boot, ggplot2, tableone, and pROC. This analysis included covariates with a *p*-value < 0.05 in univariate analysis. A two-sided *p*-value of *p* < 0.05 was considered statistically significant.

## 3. Results

### 3.1. Enrolled patients and baseline characteristics

**Figure 1.**
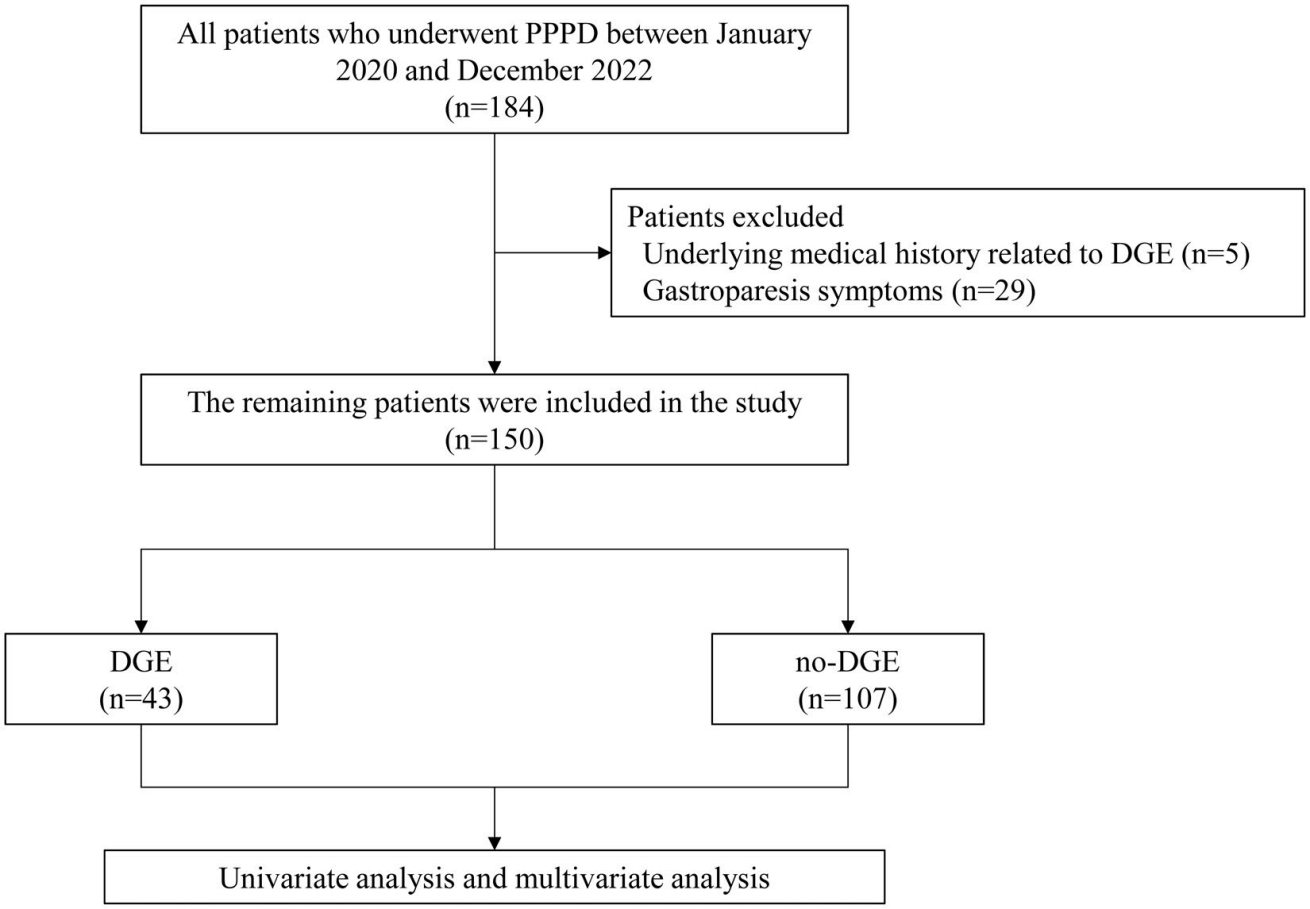
The flowchart of patient selection.

The data of patients with pancreatic head cancer, ampullary tumor, and other diseases requiring PPPD at our hospital between January 2020 and December 2022 were retrospectively collected, and a total of 184 patients were initially screened, as illustrated in Figure 1. After excluding 34 patients with a history of conditions that could induce DGE or those who manifested clinical signs of DGE prior to surgical intervention, 150 patients were included in the analysis. Patients were assigned to the DGE group and the non-DGE group according to the occurrence of postoperative gastroparesis. The DGE group comprised 43 patients, including 24 males and 19 females, with a median age of 66 (43–79) years. The non-DGE group consisted of 107 patients, including 67 males and 40 females, with a median age of 65 (41–80) years. The overall incidence of DGE was 28.67%. Baseline characteristics (Table 1) revealed no significant associations between the occurrence of DGE post-PPPD and gender, age, history of preoperative smoking or alcohol consumption, diabetes, previous surgeries, and preoperative biliary drainage (*p* > 0.05). However, patients in the DGE group had a higher BMI compared to the non-DGE group (*p* = 0.013), indicating that patients with a high BMI may be more likely to develop DGE after PPPD. Similarly, the proportion of patients with hypertension was significantly higher in the DGE group (46.51% vs 28.97%, *p* = 0.040), indicating that hypertension may also be a risk factor for DGE. After assessment using HAMA revealed that the proportion of patients with anxiety was higher in the DGE group compared to the non-DGE group (72.09% vs 48.60%, *p* = 0.009), indicating that patients with preoperative anxiety were more likely to develop DGE postoperatively.

**Table 1.** Baseline data of patients in delayed gastric emptying group and non-delayed gastric emptying group.

Characteristic	DGE	non-DGE	*p*-value
Total number of patients	43	107	
Gender			0.441
Male (%)	24 (55.81)	67 (62.62)	
Female (%)	19 (44.19)	40 (37.38)	
Age	66.00 (58.00, 70.00)	65.00 (59.00, 70.00)	0.832
BMI	23.50 ± 3.85	22.20 ± 2.39	0.013*
Smoking (%)	19 (44.19)	48 (44.86)	0.940
Alcohol consumption (%)	3 (6.98)	18 (16.82)	0.116
Hypertension (%)	20 (46.51)	31 (28.97)	0.040*
Diabetes (%)	6 (13.95)	23 (21.50)	0.290
Prior surgical history (%)	20 (46.51)	48 (44.86)	0.854
Preoperative biliary drainage (%)	6 (13.95)	18 (16.82)	0.665
Anxiety state (%)	31 (72.09)	52(48.60)	0.009*

DGE: delayed gastric emptying group; BMI: body mass index.

### 3.2. Comparison of laboratory indicators before and after operation

All patients underwent statistical analysis of laboratory indicators, and the detailed results are summarized in Table 2. The analysis revealed no significant differences in preoperative hemoglobin levels, tumor marker levels, and postoperative hemoglobin levels on the 1st, 3rd, and 5th day between the two groups (*p* > 0.05), indicating that these variables may not significantly impact the development of DGE. Conversely, the median preoperative blood lipid level was significantly higher in the DGE group (1.87 (1.30, 2.36) mmol/L) compared to the non-DGE group (1.39 (0.96, 2.02) mmol/L) (*p* = 0.005), indicating that preoperative blood lipid levels may influence the development of DGE. However, no significant difference in cholesterol levels was noted between the two groups (*p* = 0.840), indicating that cholesterol did not affect the development of gastroparesis. Likewise, no significant differences in preoperative total bilirubin, preoperative direct bilirubin, preoperative albumin, and albumin levels on postoperative days 1 and 5 were noted between the two groups (*p* > 0.05); nonetheless, this does not signify that preoperative liver dysfunction does not influence the onset of DGE. Of note, albumin levels were significantly lower in the DGE group (33.18 ± 4.31 g/L) compared to the non-DGE group (35.23 ± 3.47 g/L) on the third day post-surgery (*p* = 0.003), suggesting that postoperative hypoproteinemia resulting from traumatic surgery may influence the occurrence of DGE.

**Table 2.** Comparison of laboratory indicators between delayed gastric emptying group and non-delayed gastric emptying.

Characteristic	DGE	non-DGE	*p*-value
Preoperative hemoglobin (g/L)	123.44 ± 16.65	122.52 ± 18.87	0.781
Preoperative triglyceride (mmol/L)	1.87 (1.30, 2.36)	1.39 (0.96, 2.02)	0.005*
Preoperative cholesterol (mmol/L)	4.11 (3.59, 5.28)	4.31 (3.65, 5.23)	0.840
Preoperative total bilirubin (μmol/L)	85.20 (15.10, 177.30)	30.60 (11.80, 161.40)	0.148
Preoperative direct bilirubin (μmol/L)	77.90 (6.60, 157.40)	19.80 (5.00, 140.90)	0.105
Preoperative albumin (g/L)	40.52 ± 5.69	40.67 ± 4.98	0.871
Hemoglobin on postoperative day 1 (g/L)	118.02 ± 18.50	122.13 ± 15.92	0.175
Albumin on postoperative day 1 (g/L)	31.97 ± 4.29	33.38 ± 4.07	0.061
Hemoglobin on postoperative day 3 (g/L)	101.14 ± 12.91	103.07 ± 16.36	0.489
Albumin on postoperative day 3 (g/L)	33.18 ± 4.31	35.23 ± 3.47	0.003*
Hemoglobin on postoperative day 5 (g/L)	103.47 ± 12.86	106.59 ± 14.54	0.221
Albumin on postoperative day 5 (g/L)	34.05 ± 4.25	35.04 ± 4.32	0.202

DGE: delayed gastric emptying.

### 3.3. Comparison of surgical characteristics, clinical staging, and postoperative complications

All patients underwent radical, open PPPD, and operative characteristics were compared between the two groups (Table 3). Operative time, intraoperative blood loss volume, intraoperative blood transfusion volume, and disease type were comparable between the two groups (*p* > 0.05).

**Table 3.** Comparison of surgical characteristics, and postoperative complications between delayed gastric emptying group and non-delayed gastric emptying group.

Characteristic	DGE	non-DGE	*p*-value
Operative time (min)	255.74 ± 52.89	259.98 ± 63.21	0.698
Intraoperative blood loss (mL)	300.00 (200.00, 400.00)	300.00 (200.00, 400.00)	0.670
Intraoperative blood transfusion (%)	3 (6.98)	13 (12.15)	0.353
Disease type			0.935
pancreatic head cancer (%)	16 (37.21)	43 (40.19)	
Duodenal adenocarcinoma (%)	14 (32.56)	30 (28.04)	
Bile duct adenocarcinoma (%)	8 (18.60)	19 (17.76)	
Other diseases (%)	5 (11.63)	15 (14.02)	
Postoperative pancreatic fistula (%)	19 (44.19)	36 (33.64)	0.226
Postoperative bile leakage (%)	0 (0.00)	5 (4.67)	0.348
Postoperative bleeding (%)	4 (9.30)	8 (7.48)	0.968
Postoperative abdominal infection (%)	9 (20.93)	16 (14.95)	0.374

DGE: delayed gastric emptying.

### 3.4. Firth penalized logistic regression analysis

**Figure 2.**
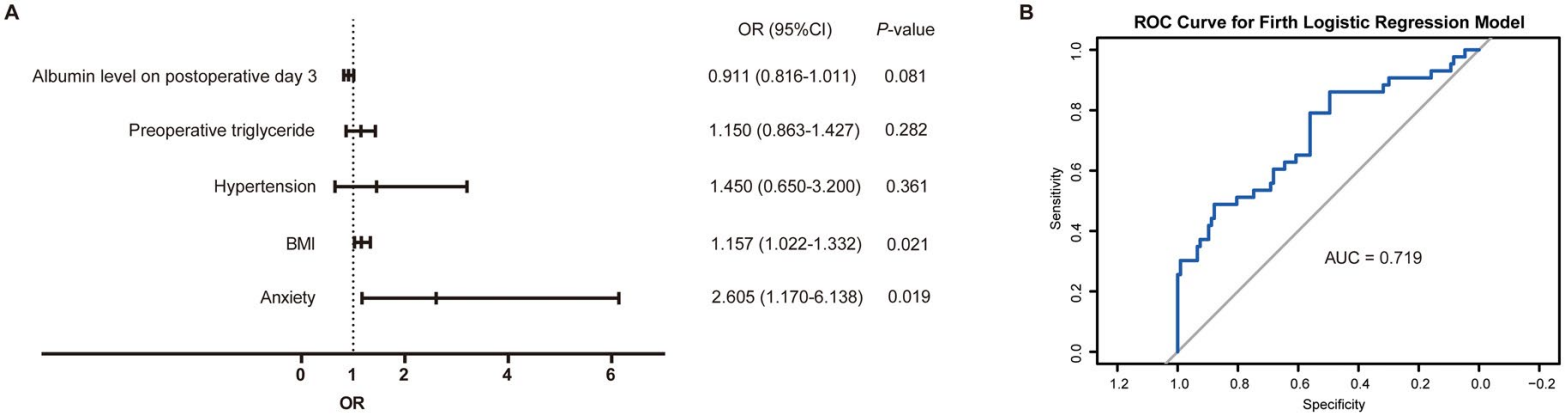
** ** A. Forest Plot of multivariate analysis of risk factors for delayed gastric emptying after pylorus-preserving pancreaticoduodenectomy. B. The predictive performance test of the firth penalized logistic regression model.

Subsequently, factors with statistical differences between the two groups were incorporated into a Firth penalized logistic regression analysis to identify risk factors for DGE, and the results are detailed in Table 4 and Figure 2A. Collinearity diagnostics of the regression model (Table S1) revealed a mean variance inflation factor (VIF) of 1.103, indicating no severe multicollinearity among the independent variables in the model. The receiver operating characteristic (ROC) curve analysis demonstrated that the area under the curve (AUC) for predicting the occurrence of DGE using this regression model was 0.719 (Figure 2B), suggesting good predictive performance. The results uncovered that the OR value of the anxiety state was 2.605 (95%CI was 1.170–6.138), indicating that preoperative anxiety state was independently related to a higher risk of DGE (*p* = 0.019). Similarly, the OR value of BMI was 1.157 (95%CI: 1.022–1.332), indicating that preoperative BMI was positively correlated with the risk of DGE (*p* = 0.021). On the other hand, other factors were not identified as independent risk factors for DGE.

**Table 4.** Firth penalized logistic regression analysis of risk factors for delayed gastric emptying after pylorus-preserving pancreaticoduodenectomy.

Characteristic	OR	95% CI	*p*-value
BMI	1.157	1.022–1.332	0.021*
Hypertension	1.450	0.650–3.200	0.361
Anxiety state	2.605	1.170–6.138	0.019*
Preoperative triglyceride	1.150	0.863–1.427	0.282
Albumin level on postoperative day 3	0.911	0.816–1.011	0.081

BMI: body mass index.

### 3.5. Analysis of the correlation between risk factors and the severity of DGE

Due to the limited number of patients included in this study, not all factors affecting the severity of DGE were analyzed in order to avoid increasing the risk of bias. Only the risk factors mentioned above were evaluated. Among the 43 patients with DGE, 18 were classified as Grade A, 16 as Grade B, and 9 as Grade C. Given the small number of patients in Grade C (only 9 cases), patients classified as Grade B and Grade C were combined into one group for analysis to enhance the reliability of the results. The findings (Table 5) indicated that there was no correlation between the aforementioned risk factors and the severity of DGE (*p* > 0.05).

**Table 5.** Analysis of the correlation between risk factors and the severity of DGE.

Characteristic	DGE grade A	DGE grade B/C	*p*-value
Total number of patients	18	25	
BMI	22.94 (20.83, 25.33)	23.03 (21.48, 24.97)	0.844
Hypertension (%)	7 (38.89)	13 (52.00)	0.395
Anxiety state (%)	11 (61.11)	20 (80.00)	0.173
Preoperative triglyceride	1.83 (0.98, 2.42)	2.04 (1.54, 2.35)	0.546
Albumin level on postoperative day 3	33.28 ± 3.71	33.10 ± 4.77	0.898

DGE: delayed gastric emptying group; BMI: body mass index.

### 3.6. Comparison between the two groups after PSM

A 1:1 PSM was performed (including the following normalized covariates: age, gender, and POPF). The efficacy of PSM is presented in Table S2. Subsequently, a further analysis of the aforementioned risk factors for DGE was conducted. The results (Table 6) revealed a statistically significant difference in anxiety state (*p* = 0.042) between the two groups, indicating a strong association between anxiety state and DGE. Conversely, no significant differences were observed in other preoperative data, surgical characteristics, laboratory indicators, or postoperative complications after PSM, as detailed in Table S3.

**Table 6.** Comparison of risk factors between delayed gastric emptying group and non-delayed gastric emptying group after propensity score matching.

Characteristic	DGE	non-DGE	*p*-value
Total number of patients	42	42	
BMI	22.94 (21.07, 25.10)	22.55 (20.80, 24.15)	0.209
Hypertension (%)	19 (45.24)	16 (38.10)	0.507
Anxiety state (%)	31 (73.81)	22 (52.38)	0.042*
Preoperative blood lipids (mmol/L)	1.87 (1.35, 2.36)	1.33 (1.00, 2.30)	0.087
Albumin 3 days after surgery (g/L)	33.22 ± 4.36	34.73 ± 3.81	0.096

DGE: delayed gastric emptying.

## 4. Discussion

Through retrospective analysis, the present study unveiled that risk factors for DGE after PPPD included anxiety state, BMI, hypertension, preoperative blood lipid levels, and albumin levels on the third day after surgery. Additionally, anxiety and BMI were identified as independent risk factors for DGE. In contrast to previous studies, this study noted the influence of psychological factors on clinical outcomes. Noteworthily, to the best of our knowledge, this is the first study to analyze the correlation between anxiety state and DGE post-PPPD. As anticipated, the presence of a preoperative anxiety state increased the risk of DGE after PPPD, though it did not affect the severity of DGE. This finding provides a theoretical reference for psychological intervention in PPPD patients with anxiety symptoms, which may assist in postoperative recovery and DGE prevention. It also provides theoretical support for subsequent multi-center, large-sample randomized clinical trials.

With the development of the biopsychosocial medical model, the impact of psychological state on disease treatment and prognosis has garnered extensive attention. As previously noted, while anxiety is a prevalent psychological condition among cancer patients [13], psychological tolerance varies among patients. Some patients may experience severe psychological distress at the time of diagnosis, whereas may develop psychological disorders secondary to the trauma imposed by surgical modalities. Therefore, whether psychological conditions impact treatment efficacy and prognosis, or whether clinical treatment induces psychological disorders, warrants further investigation. Prior investigations have reported that anxiety is closely related to gastrointestinal dysfunction in patients with functional dyspepsia [22,23]. In addition, anxiety can also affect gastric absorption function in patients undergoing surgery [24]. This study examined the effect of preoperative anxiety state on the incidence of postoperative DGE, and the results showed that the presence of preoperative anxiety state increased the risk of DGE after PPPD. Furthermore, factors identified through univariate analysis were incorporated into the multivariate logistic regression analysis, revealing that anxiety state was an independent risk factor for DGE after PPPD. In other words, preoperative psychological disturbances can influence the incidence of postoperative complications. Recent studies have pointed out that the severity of anxiety symptoms is correlated with the degree of gastroparesis [25]. According to an earlier study, psychological interventions can alleviate negative emotions in patients following abdominal surgery, thereby reducing the duration of DGE symptoms [26]. In addition, previous studies have documented that the occurrence of postoperative anxiety and depression may be caused by the prolonged disease course and the presence of postoperative complications [27]. Therefore, psychological factors cannot be overlooked, and their presence may lead to various complications, delay postoperative recovery, and affect the quality of life of patients. Thus, timely and appropriate psychological interventions should be considered in patients undergoing PPPD.

Studies have shown that among gastric cancer patients who underwent gastrectomy, obese patients are more likely to develop DGE compared to non-obese patients [28]. Glucagon-like peptide-1 (GLP-1) is a hormone secreted postprandially that stimulates insulin secretion. Obesity increases GLP-1 levels and concurrently decreases gastric motility. Meanwhile, PD also promotes GLP-1 secretion. The combined effect of obesity and PD may significantly increase the risk of DGE [29,30]. BMI, the most commonly adopted index for evaluating obesity, has been associated with the risk of DGE after PPPD, with higher BMI correlating with a higher risk of DGE [12,31–33]. Herein, univariate analysis indicated that patients with high BMI were more likely to develop DGE after PPPD compared with patients with low BMI, which is consistent with the findings of previous studies. In subsequent multivariate analysis, BMI was identified as an independent risk factor for DGE after PD, with higher BMIs associated with a higher risk of DGE. This observation may be attributed to abdominal fat accumulation increasing surgical difficulty and prolonged operative time, which collectively contribute to increased tissue exposure time, and intraoperative trauma, thereby increasing the risk of DGE [34]. Numerous studies have identified no significant association between obesity and long-term follow-up outcomes of patients after PD [34–36]. These discrepancies may be due to regional variations, differences in surgical skills, and racial heterogeneity. However, clinicians should be vigilant regarding the increased risk of DGE in overweight patients following PPPD and accordingly implement prompt interventions.

Several studies have identified a close association between obesity and abnormal blood lipid levels [37–39]. However, herein, not all patients with a BMI exceeding 25 presented with hyperlipidemia, whereas some patients with a BMI below 20 had hyperlipidemia. Nonetheless, it is worth acknowledging that lipid levels were higher in obese patients than in non-obese patients. In the present study, dyslipidemia was identified as a risk factor for DGE after PPPD, and patients with higher lipid levels had a greater risk of DGE. Although no study has investigated the relationship between hyperlipidemia and the risk of DGE after PPPD, previous studies have documented that hyperlipidemia may increase the risk of postoperative complications and reduce the quality of life of patients after PD [40–43]. Therefore, it can be inferred that preoperative lipid-lowering strategies to maintain blood lipid levels within the normal range may mitigate the risk of DGE after PPPD.

Hypertension, as one of the common chronic diseases, was also established as a risk factor for DGE herein. Two previous retrospective studies have yielded conflicting results; one study [44] suggested that hypertension was positively correlated with the risk of DGE after pancreaticoduodenectomy with or without pylorus preservation, whereas the other study [45] identified no correlation between hypertension and the risk of DGE following PD. These two studies, as well as this study, were limited by small sample sizes and retrospective designs. Given that retrospective studies are observational, they are prone to confounding biases, which may compromise the validity of the results. Therefore, multicenter, large-sample prospective studies are necessitated to obtain reliable evidence and guide clinical decision-making.

Malnutrition in pancreatic head cancer patients undergoing PD treatment can increase the risk of postoperative complications, which is closely related to adverse short-term outcomes [46]. In a retrospective study incorporating clinical trial data, Martín Santos et al. noted that preoperative hypoproteinemia was an independent risk factor for DGE [47]. In this study, although the results confirmed that the preoperative albumin level did not influence the incidence of DGE, albumin levels on the third postoperative day could serve as a protective factor against the development of DGE after PPPD. Serum albumin is an indicator of the nutritional status of patients [11], which is closely related to their quality of life. Thus, improvements in nutritional status facilitate postoperative recovery, thereby enhancing immune resistance and reducing the risk of complications. A prospective study outlined that at 24 months after PD, patients in the non-DGE group had significantly higher serum albumin levels than those in the DGE group [10]. In addition, patients who developed DGE following gastric cancer surgery also had significantly lower serum albumin levels than those without DGE [48]. Taken together, these studies corroborate the results of the present study. Therefore, appropriate albumin supplementation or early nutritional support after PD can not only accelerate postoperative recovery but also mitigate the risk of DGE.

Studies have demonstrated that age, sex, and POPF are closely associated with the occurrence of DGE [31,49]. To validate the reliability of the present findings, PSM was further performed. The results revealed a statistically significant difference in anxiety state between the two groups. This observation indicates that anxiety symptoms remain an independent risk factor for DGE after adjusting for currently known key confounding variables.

However, some limitations of this study cannot be overlooked. To begin, this was a single-center, small-sample retrospective study, which may limit the generalizability of the findings. It only serves as a reference for subsequent large-sample, multi-center randomized controlled trials. Secondly, Chen et al. [28] identified visceral obesity as an independent risk factor for DGE following subtotal gastrectomy. However, in this study, only BMI data were collected, and no data related to visceral obesity were collected. Thirdly, this study only recorded the preoperative anxiety scores of patients and analyzed the incidence of complications (DGE) at our center.

In summary, anxiety state, high BMI, hypertension, high preoperative lipid levels or hyperlipidemia, and lower albumin levels on the third postoperative day were associated with an increased risk of DGE after PPPD. Moreover, anxiety state was identified as an independent risk factor for DGE. These findings collectively highlight the need for implementing timely and appropriate psychological interventions in patients undergoing PPPD treatment.

## Supplementary Material

Table S1.docx

STROBE_checklist.doc

Table S2.docx

Table S3.docx

## Data Availability

The datasets analyzed during the current study are available from the corresponding author on reasonable request.
