# Supplementary material for: Preoperative anxiety state is an independent risk factor for delayed gastric emptying after pylorus-preserving pancreaticoduodenectomy: a single-center retrospective case-control study
Source: Ann Med. 2025 Sep 23;57(1):2564278. doi: 10.1080/07853890.2025.2564278 (PMC12462420; doi:10.1080/07853890.2025.2564278)
Supplement: Table S1.docx [file IANN_A_2564278_SM3038.docx]

Table S1 Collinearity tests of Firth penalized logistic regression models

| Characteristic | VIF |
| --- | --- |
| Hypertension (as.numeric) | 1.119 |
| Preoperative triglyceride | 1.022 |
| Albumin level on postoperative day 3 | 1.196 |
| Anxiety state (as.numeric) | 1.075 |
| Mean value | 1.103 |

Abbreviations: VIF, variance inflation factor
