# Supplementary material for: Preoperative anxiety state is an independent risk factor for delayed gastric emptying after pylorus-preserving pancreaticoduodenectomy: a single-center retrospective case-control study
Source: Ann Med. 2025 Sep 23;57(1):2564278. doi: 10.1080/07853890.2025.2564278 (PMC12462420; doi:10.1080/07853890.2025.2564278)
Supplement: Table S2.docx [file IANN_A_2564278_SM3036.docx]

Table S2 Comparison of baseline data between the two groups before and after propensity score matching

| Characteristic | Before PSM | | | | After PSM | | | |
| --- | --- | --- | --- | --- | --- | --- | --- | --- |
|  | DGE | non-DGE | *P*-value | SMD | DGE | non-DGE | *P*-value | SMD |
| Age |  |  | 0.392 |  |  |  | 1.000 |  |
| <65 | 16 (37.21) | 48 (44.86) |  | -0.158 | 16 (38.10) | 16 (38.10) |  | 0.000 |
| ≥65 | 27 (62.79) | 59 (55.14) |  | 0.158 | 26 (61.90) | 26 (61.90) |  | 0.000 |
| Gender |  |  | 0.441 |  |  |  | 1.000 |  |
| Male | 24 (55.81) | 67 (62.62) |  | -0.137 | 24 (57.14) | 24 (57.14) |  | 0.000 |
| Female | 19 (44.19) | 40 (37.38) |  | 0.137 | 18 (42.86) | 18 (42.86) |  | 0.000 |
| Postoperative pancreatic fistula |  |  | 0.392 |  |  |  | 1.000 |  |
| No (%) | 24 (55.81) | 71 (66.36) |  | -0.158 | 24 (57.14) | 24 (57.14) |  | 0.000 |
| Yes (%) | 19 (44.19) | 36 (33.64) |  | 0.158 | 18 (42.86) | 18 (42.86) |  | 0.000 |

Abbreviations: DGE, delayed gastric emptying; PSM, propensity score matching
