# Supplementary material for: Preoperative anxiety state is an independent risk factor for delayed gastric emptying after pylorus-preserving pancreaticoduodenectomy: a single-center retrospective case-control study
Source: Ann Med. 2025 Sep 23;57(1):2564278. doi: 10.1080/07853890.2025.2564278 (PMC12462420; doi:10.1080/07853890.2025.2564278)
Supplement: Table S3.docx [file IANN_A_2564278_SM3034.docx]

Table S3 Comparison between delayed gastric emptying group and non-delayed gastric emptying group after propensity score matching

| Characteristic | DGE | non-DGE | *P*-value |
| --- | --- | --- | --- |
| Total number of patients | 42 | 42 |  |
| Gender |  |  | 1.000 |
| Male (%) | 24 (57.14) | 24 (57.14) |  |
| Female (%) | 18 (42.86) | 18 (42.86) |  |
| Age | 66.00 (58.50, 69.75) | 65.50 (58.25, 71.00) | 0.982 |
| BMI | 22.94 (21.07, 25.10) | 22.55 (20.80, 24.15) | 0.209 |
| Smoking (%) | 19 (45.24) | 20 (47.62) | 0.827 |
| Drinking (%) | 3 (7.14) | 7 (16.67) | 0.178 |
| Hypertension (%) | 19 (45.24) | 16 (38.10) | 0.507 |
| Diabetes mellitus (%) | 6 (14.29) | 8 (19.05) | 0.558 |
| Previous surgical history (%) | 20 (47.62) | 19 (45.24) | 0.827 |
| Preoperative biliary drainage (%) | 5 (11.90) | 10 (23.81) | 0.154 |
| Anxiety state (%) | 31 (73.81) | 22 (52.38) | 0.042* |
| Preoperative hemoglobin (g/L) | 123.64±16.80 | 118.79±20.65 | 0.240 |
| Preoperative blood lipids (mmol/L) | 1.87 (1.35, 2.36) | 1.33 (1.00, 2.30) | 0.087 |
| Preoperative cholesterol (mmol/L) | 4.11 (3.61, 5.25) | 4.34 (3.63, 5.56) | 0.494 |
| Preoperative total bilirubin (μmol/L) | 87.45 (15.38, 174.30) | 33.30 (11.20, 160.30) | 0.180 |
| Preoperative direct bilirubin (μmol/L) | 79.50 (6.92, 155.50) | 27.70 (4.85, 138.82) | 0.175 |
| Preoperative albumin (g/L) | 40.58±5.75 | 40.83±5.49 | 0.839 |
| Hemoglobin 1 day after surgery (g/L) | 121.00 (105.25, 131.00) | 118.50 (109.25, 134.00) | 0.840 |
| Albumin 1 day after surgery (g/L) | 31.95±4.34 | 32.92±4.16 | 0.300 |
| Hemoglobin 3 days after surgery (g/L) | 101.21±13.06 | 101.36±18.38 | 0.967 |
| Albumin 3 days after surgery (g/L) | 33.22±4.36 | 34.73±3.81 | 0.096 |
| Hemoglobin 5 days after surgery (g/L) | 103.57±12.99 | 104.79±15.21 | 0.695 |
| Albumin 5 days after surgery (g/L) | 34.10±4.28 | 35.29±5.00 | 0.247 |
| Operation time (min) | 256.12±53.47 | 262.50±59.06 | 0.605 |
| Intraoperative blood loss (mL) | 300.00 (200.00, 400.00) | 400.00 (300.00, 400.00) | 0.125 |
| Intraoperative blood transfusion (%) | 3 (7.14) | 7 (16.67) | 0.178 |
| Disease type |  |  | 0.579 |
| pancreatic head cancer (%) | 16 (38.10) | 17 (40.48) |  |
| Duodenal adenocarcinoma (%) | 13 (30.95) | 9 (21.43) |  |
| Bile duct adenocarcinoma (%) | 8 (19.05) | 7 (16.67) |  |
| Other diseases (%) | 5 (11.90) | 9 (21.43) |  |
| Postoperative pancreatic fistula (%) | 18 (42.86) | 18 (42.86) | 1.000 |
| Postoperative bile leakage (%) | 0 (0.00) | 2 (4.76) | 0.474 |
| Postoperative bleeding (%) | 4 (9.52) | 5 (11.90) | 1.000 |
| Postoperative abdominal infection (%) | 8 (19.05) | 5 (11.90) | 0.365 |

Abbreviations: DGE, delayed gastric emptying
